# HSP70 regulates Eg5 distribution within the mitotic spindle and modulates the cytotoxicity of Eg5 inhibitors

**DOI:** 10.1038/s41419-020-02919-7

**Published:** 2020-09-01

**Authors:** Chieh-Ting Fang, Hsiao-Hui Kuo, Shao-Chun Hsu, Ling-Huei Yih

**Affiliations:** 1 Department of Life Science, National Taiwan University, Taipei, Taiwan; 2 Institute of Cellular and Organismic Biology, Academia Sinica, Taipei, Taiwan

**Keywords:** Mitosis, Kinesin, Chaperones

## Abstract

The heat shock protein 70 (HSP70) is a conserved molecular chaperone and proteostasis regulator that protects cells from pharmacological stress and promotes drug resistance in cancer cells. In this study, we found that HSP70 may promote resistance to anticancer drugs that target the mitotic kinesin, Eg5, which is essential for assembly and maintenance of the mitotic spindle and cell proliferation. Our data show that loss of HSP70 activity enhances Eg5 inhibitor-induced cytotoxicity and spindle abnormalities. Furthermore, HSP70 colocalizes with Eg5 in the mitotic spindle, and inhibition of HSP70 disrupts this colocalization. Inhibition or depletion of HSP70 also causes Eg5 to accumulate at the spindle pole, altering microtubule dynamics and leading to chromosome misalignment. Using ground state depletion microscopy followed by individual molecule return (GSDIM), we found that HSP70 inhibition reduces the size of Eg5 ensembles and prevents their localization to the inter-polar region of the spindle. In addition, bis(maleimido)hexane-mediated protein-protein crosslinking and proximity ligation assays revealed that HSP70 inhibition deregulates the interaction between Eg5 tetramers and TPX2 at the spindle pole, leading to their accumulation in high-molecular-weight complexes. Finally, we showed that the passive substrate-binding activity of HSP70 is required for appropriate Eg5 distribution and function. Together, our results show that HSP70 substrate-binding activity may regulate proper assembly of Eg5 ensembles and Eg5-TPX2 complexes to modulate mitotic distribution/function of Eg5. Thus, HSP70 inhibition may sensitize cancer cells to Eg5 inhibitor-induced cytotoxicity.

## Introduction

The mitotic spindle is a self-organized structure composed of numerous microtubules (MTs), MT-associated proteins (MAPs), and motor proteins. The tightly regulated assembly of this structure during mitosis ensures faithful chromosome segregation and prevents the occurrence of genomic instability that is linked to the tumorigenesis and cancer drug resistance^1^. From the onset of mitosis to metaphase, two centrosomes migrate to opposite sides of the cell and function as spindle poles. MTs are arranged with minus ends focused at the spindle poles and plus ends extended toward the chromosomes in the middle region of the spindle; this arrangement defines the bipolarity of a mitotic spindle^2^. In a typical metaphase spindle, four categories of MTs exist: kinetochore-MTs (k-MTs), which have plus ends directly connected to the kinetochore; astral MTs, which grow away from poles toward the cell cortex; spindle MTs, which grow away from poles toward the chromosome but are not connected to the kinetochores; and interdigitating MTs, which emanate from the two opposite poles and overlap in an antiparallel manner at the spindle midzone^3^. Importantly, the coordinated organization of a bipolar spindle involves the continuous growth and shrinkage of the MTs. This property, called dynamic instability, is driven by the concerted actions of MTs, MAPs and MT motors^4^, and as such, it requires intricate protein quality control.

The HSP70 is a highly conserved family of molecular chaperone that plays central role in cellular proteostasis by aiding the folding/refolding, oligomerization, and turnover of client proteins^5^, which collectively serve to protect cells from physiological, pathological, environmental, and pharmacological insults and render HSP70 a critical determinant of cancer cell resistance against therapeutics^6,7^. Among the six HSP70 members in the human cytosol/nucleus, the HSP70-1L, HSP70.2, and HSP70-6 proteins (encoded by *HSPA1L*, *HSPA2* and *HSPA6*, respectively) have been demonstrated to be expressed in specific tissues including testes, brain, and blood but nearly undetectable in other cell types^6,8,9^. Among the other three members, by RNAi or antibodies that specifically targeting or recognizing HSP70-1A and −1B (encoded by *HSPA1A/1B*, hereafter HSP70) but not HSC70 (encoded by *HSPA8*), we and others have previously demonstrated in human mitotic cells that HSP70 localizes to the mitotic centrosome under the control of multiple mitotic kinases after nuclear envelope breakdown. This centrosome localization of HSP70 is required for proper centrosome function, MT nucleation, MT dynamics, and mitotic spindle assembly^10–13^. However, the mechanism by which HSP70 modulates MT dynamics and mitotic spindle assembly remains unclear.

Kinesin-5 (Eg5 in human cells) is one of several plus end-directed kinesin motors that play essential roles in MT dynamics and bipolar spindle assembly and maintenance. It has been recognized as a valuable drug target for anticancer therapies and several classes of Eg5 inhibitors have been developed^14^. Monastrol, ispinesib and (+)-*S*-Trityl-L-cysteine (TriC) are the loop-5 inhibitors and can weaken Eg5 binding to MTs, disrupting Eg5-dependent outward force production and inducing the formation of monopolar spindles with unseparated spindle poles^14–19^. BRD9876 (BRD) is the “rigor” inhibitor that locks Eg5 in a state with enhanced MT binding, leading to bundling and stabilization of MTs^20,21^. The differential effects induced by these inhibitors implied multiple mitotic functions of Eg5 within the spindle. However, cultured cancer cells have been observed to develop resistance to Eg5 inhibitors, limiting clinical applications of these drugs^14,22–24^. To make improvements in cancer treatments targeting Eg5, a comprehensive understanding of the multiple functions of Eg5 and how these functions are modulated during spindle assembly is crucial.

The Eg5 protein contains a motor head domain, a stalk domain and a tail domain, and it assembles into a bipolar homotetramer with a pair of motor heads at each opposing side of the complex^4,25,26^. As implied from studies performed in various model systems, this organization allows Eg5 tetramers to engage two adjacent MTs and participate in critical functions during spindle assembly. For example, the bipolar homotetrameric conformation of Eg5 and formation of monopolar spindles after Eg5 inactivation imply that Eg5 slides antiparallel MTs apart at the spindle midzone and contributes to centrosome separation^15,16,27–33^. Recent studies using in vitro assays and xenopus spindle extracts revealed that Eg5 can also crosslink parallel MTs and promotes MT bundling and stabilization near the spindle poles^21,34–36^. Eg5-mediated MT crosslinking may also be important for controlling k-MT bundling into k-fibers and k-fiber incorporation into the spindle to facilitate MT-chromosome attachment^2,37–39^. Notably, Eg5 phosphorylation at several sites has been speculated to spatiotemporally modulate Eg5 functions during mitosis^40–43^. In addition, the motility, directionality and localization of Eg5 on MT can be modulated by the interactions among Eg5 tetramers or between Eg5 and other MAPs or MT motors such as TPX2, kinesin-12 or dynein^23,37,44–51^. It was proposed that these interactions can lead to formation of Eg5 tetramers-TPX2-dynein-containing protein complexes that undergo dynamic disassembly/reassembly^51^, implying the involvements of the proteostasis regulations of Eg5 motility and localization within the spindle. However, the regulation of Eg5 distribution within different MT pools or different spindle regions has not been demonstrated in mammalian cells.

Previously, we showed that pharmacological inhibition of HSP70 disrupts spindle assembly to induce mitotic arrest and cell death^10^. Since HSP70 in yeast is known to regulate the Eg5 homologue, Cin8, and spindle length^52^, we asked whether HSP70 modulates the mitotic functions of Eg5 and cellular sensitivity to Eg5 inhibitors in human cells. We found that HSP70 interacts with Eg5 and is required for accurate Eg5 function and localization within the spindle, thus ensuring maintenance of spindle integrity. Based on these molecular actions, HSP70 expression and activity are important determinants of cellular sensitivity to Eg5 inhibitor-induced spindle abnormalities and cell death.

## Materials and methods

### Cell culture and drug treatments

CGL2 cells (a HeLa cell/normal human fibroblast hybrid) and HeLaS3 cells were cultured as previously described^10,11^. To synchronize the cell cycle at mitosis, CGL2 cells were subjected to a double-thymidine block and were released from the block for 9.5 h^10^. HSP70 inhibitors include pifithrin-μ (PES, No. 2653, Tocris Bioscience, Bistro, UK), VER-155008 (VER, No. 17257, Cayman Chemical, Ann Arbor, MI, USA), and YM-1 (SML0943, Sigma Aldrich, St. Louis, MO, USA). Eg5 inhibitors include (+)-*S*-Trityl-L-cysteine (TriC, No. 164739, Sigma), BRD9876 (BRD, No. 5454, Tocris), and SB743921 (No. 18384, Cayman). Each drug was first tested for their cytotoxicity in each cell line by 72 h viability assay^10,11^ until the IC_50_ of each drug for each cell line is determined. Treatment concentration, which is indicated in each figure, was set to be twofold IC_50_ for 16 h overnight treatment and 3–4-fold IC_50_ for 1 h acute treatment. Except the 72 h cytotoxicity assay or those indicated in the figures, all drug treatments were made 1 h prior to examination to reveal the immediate effects on mitotic cells.

### Depletion and overexpression of HSP70

Depletion of endogenous HSP70 was accomplished using shRNAs prepared as described previously^10,11^. The shRNAs targeting *HSPA1A and HSPA1B* (TRCN-8757, −342860, −8760, and −8763) were purchased from the National RNAi Core Facility (Genomic Research Center, Academia Sinica). Cells expressing FLAG-tagged HSPA1A-wild type (WT), -Lys^71^ to Glu (K71E), -Glu^175^ to Ser (E175S) and Ala^403^ + Val^438^ to Gly [A403G + V438G, (GG)] were established as described^10,11^ and were depleted of endogenous HSP70 using shRNA targeting the 5′-untranslated region of *HSPA1A* (TRCN-342860) in the rescue experiments. Cells expressing empty vectors (pLKO.1 and pFB-Neo) were used as controls for depletion and overexpression of HSP70, as appropriate.

### Immunoprecipitation

The synchronized mitotic cells obtained as described above were lysed and the immunoprecipitation was performed as previously described^10^. HSP70 and Eg5 were immunoprecipitated by specific HSP70 antibody (SMC-100, StressMarq, Victoria, Canada) and Eg5 antibody (#627801, BioLegend, San Diego, CA, USA), respectively, and the precipitates were analyzed by western blot^10^. GAPDH was detected with anti-GAPDH (GTX100118, GeneTex, Hsinchu, Taiwan), for use as a loading control.

### Immunofluorescence staining, proximity ligation assay (PLA), and confocal microscopy

Cells seeded on coverslips were fixed and subjected to immunofluorescence staining by previously described methods^11^. The primary antibodies included anti-HSP70 (GTX104126, GeneTex or SMC-100, StressMarq), anti-Eg5 (#627801, BioLegend or ab137535, Abcam, Cambridge, UK), anti-p-T926-Eg5 (ab61104, Abcam), anti-α-tubulin (GTX112141, GeneTex or T5168, Sigma), anti-β-tubulin (T4026, Sigma), anti-acetyl-tubulin (T6793, Sigma), anti-TPX2 (PA3-16832, ThermoFisher, Waltham, MA, USA), and anti-CEP152 (ab183911, Abcam). Alexa-Fluor 488-, 568-, 633-, and 647-conjugated goat anti-mouse, as well as anti-rabbit IgG were purchased from Invitrogen (Carlsbad, CA). After staining, cells with the indicated spindle types were counted using a Zeiss Axioplan 2 Imaging MOT fluorescence microscope. Samples were subjected to confocal imaging on a Leica TCS-SP5 microscope equipped with a HCX PLAPO 63×/1.4 objective under 300 Hz scanning speed, 15–20 μm image stacks and 0.5 μm step size. The confocal image stacks were processed and maxima-projected in ImageJ for presentation.

Line-profile analysis was performed in ImageJ to examine Eg5 distribution within the spindle. Lines were drawn free-hand along the MT fibers marked by α-tubulin immunostaining; the Eg5 intensities along the lines were measured, normalized and averaged to obtain mean distribution curves. Pairwise comparisons between the Eg5 distributions were achieved by comparing the slope of lines fitted by linear regression performed on the GraphPad Prism 7.04 software.

The interactions between Eg5 and HSP70, α-tubulin, acetyl-tubulin or TPX2 were assessed by PLA as described previously^11^. Counterstaining of the spindle after the PLA was performed following the protocol provided by Sigma. A 24 × 24 μm region of interest (ROI) covering a single cell was cropped from the PLA image using ImageJ, and the PLA intensity within the ROI was measured. To assess the distributions of Eg5-TPX2, Eg5-α-tubulin, and Eg5-acetyl-tubulin interacting regions, the ratio of PLA intensities at the pole regions over the whole-cell intensity was measured with Imaris software. A sphere was created with the diameter set to be one-third of the pole-to-pole distance; this sphere covered the pole region marked by α-tubulin staining, and the PLA intensities within the sphere were divided by those within the ROI (expressed as percentage). Conditions of staining, imaging, processing, and analysis were kept consistent for each experiment, and the intensities of markers stained and imaged in repeated experiments were normalized.

### Analysis of Eg5 localization to k-MT and MT stability

Cells seeded on coverslips in a six-well plate were treated with PES or VER for 1 h. The six-well plate was then immersed in the ice-cold water for 5 min to disassemble the spindle pole MTs. Cells were then immediately fixed and immunostained for Eg5 and α-tubulin. Eg5 intensity on k-MTs was measured using Imaris, and the cells with Eg5-decorated MTs remaining at the spindle pole were counted. Cells that were fixed without exposure to ice-cold water were used as controls.

### Analysis of Eg5-mediated bipolar spindle reassembly

Cell seeded on coverslips were treated with 2.5 μM TriC for 1 h to induce the formation of monopolar spindles. The TriC-containing medium was then removed and the cells were washed twice with PBS. Cells were then immediately fixed or incubated in fresh medium for another 30 or 60 min prior to fixation. After fixation, cells were subjected to immunofluorescence staining for mitotic spindles.

### Ground state depletion microscopy followed by individual molecule return (GSDIM) superresolution image acquisition and analysis

Mitotic cells immunostained with Eg5-Alexa-Fluor 647 and α-tubulin-Alexa-Fluor 488 were subjected to GSD imaging on a Leica SR GSD superresolution system equipped with a HCX APO 100×/1.47 objective, following the procedures described previously^11^. The Eg5 staining was resolved as clusters under GSD imaging, and a wide-field image of α-tubulin as a marker of spindles was acquired. The Eg5 cluster analysis was performed with ImageJ. A mask was created to cover the spindle region by adjusting the threshold; the spindle mask was then subdivided into pole (1/4 the pole-to-pole distance) and inter-polar regions (1/2 the pole-to-pole distance; Fig. 4c). The sizes, numbers and distributions of the Eg5 clusters within these regions were then quantified using ImageJ.

### Bis(maleimido)hexane (BMH)-mediated crosslinking of Eg5-containing protein complex and western blotting

Synchronized mitotic CGL2 cells obtained as described above were treated with PES and VER for 1 h at the onset of mitosis. A mitotic shake-off was performed to collect mitotic cells, which were then suspended in a hypotonic solution (20 mM Tris-HCl, pH7.4, with 1.5 mM MgCl_2_ and 10 mM KCl) supplemented with 5 mg/mL protease and phosphatase inhibitors (Roche) and 0.5 mM BMH (ThermoFisher, #22330). Cells were then homogenized with a Dounce homogenizer, followed by shaking at 250 rpm at room temperature for 30 min. The crosslinking reaction was quenched by addition of 1 mM DTT, and after another 30 min shaking, the homogenates were lysed for western blotting^11^. Quantification of protein bands on western blots was performed using ImageJ.

## Results

### HSP70 chaperone activity modulates cell sensitivity to Eg5 inhibitors

**Fig. 1:**
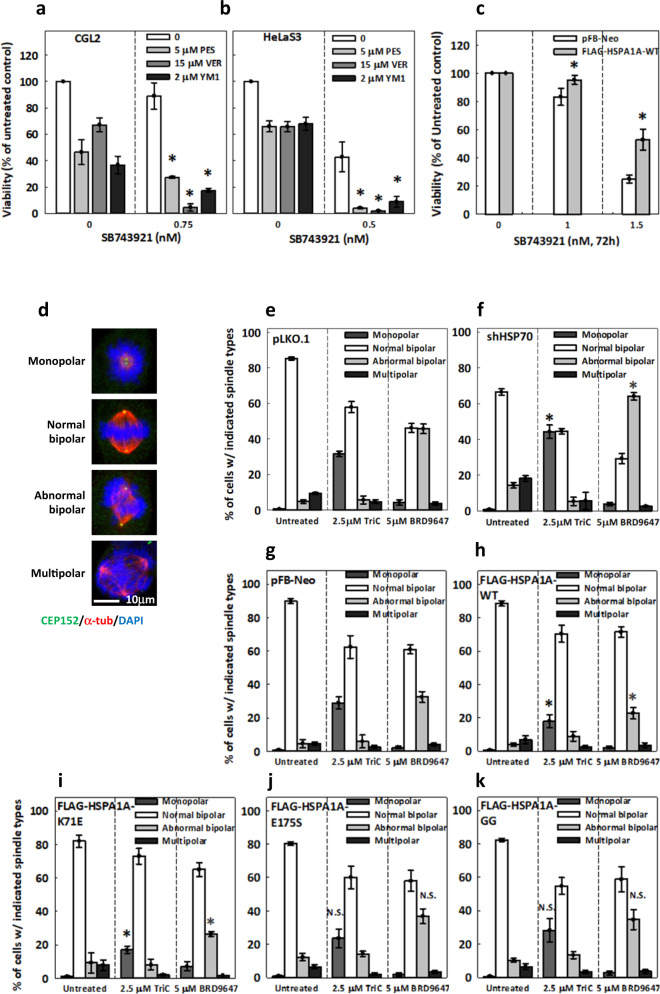
HSP70 chaperone activity attenuates cell sensitivity to Eg5 inhibitors. **a**–**b** HSP70 inhibitors enhanced the cytotoxicity of SB743921. CGL2 (**a**) and HeLaS3 (**b**) cells were incubated in medium containing SB743921 alone or in combination with HSP70 inhibitors, PES, VER or YM1, at the indicated concentrations for 72 h, after which cell viability was measured. Cell viability under HSP70 inhibitor alone was normalized to untreated cells. The mean ± SD from at least two independent experiments is shown. *p < 0.05 comparing HSP70 inhibitor-treated cells to cells without HSP70 inhibitor treatment by Student’s *t* test. **c** CGL2 cells harboring pFB-Neo empty vector or stably expressing FLAG-tagged HSP70-WT (FLAG-HSPA1A-WT) were incubated with the indicated concentration of SB743921 for 72 h before measurement of viability. Mean ± SD from at least three independent experiments is shown. *p < 0.05 comparing FLAG-HSPA1A-WT to pFB-Neo by two-way ANOVA. **d** Representative images of mitotic cells with the indicated types of spindle stained with CEP152 (green), α-tubulin (α-tub, red), and DAPI (blue). Cells were treated with 2.5 μM TriC or 5 μM BRD for 1 h and then fixed and immunostained to reveal mitotic spindles. **e**–**k** Percentages of cells with the indicated types of spindle are shown for the knock-down control (**e**, pLKO.1), HSP70-depleted (**f**, shHSP70), pFB-Neo-harboring (**g**), and FLAG-HSP70-WT (**h**, FLAG-HSPA1A-WT) and mutant-expressing (**i**–**k**) mitotic cells treated as indicated. At least 600 mitotic cells were counted for each experiment. The mean ± SD from at least three independent experiments is shown. *p < 0.05 comparing the respective spindle types and treatments between pLKO.1 and shHSP70 or between pFB-Neo and FLAG-HSPA1As by Student’s *t* test; N.S. no significance.

We first addressed whether HSP70 modulates Eg5 inhibition-induced cytotoxicity in proliferating cells by treating them with a combination of SB743921 and HSP70 inhibitors for 72 h. SB743921, a second-generation ispinesib analogue, is a potent Eg5 inhibitor under phase I/II clinical trials with reported cellular resistance^19,24^. We found in CGL2 cells and in the HeLaS3 cancer cell line that SB743921-induced cytotoxicity was enhanced by co-treatment with any of several HSP70 inhibitors, including pifithrin-μ (PES), VER-155008 (VER), and YM-1 (Fig. 1a and b). In addition, we observed that cells overexpressing FLAG-tagged HSP70 (FLAG-HSPA1A-WT) were more resistant to SB743921 treatment than the cells harboring pFB-Neo empty vector (Fig. 1c). Next, we examined mitotic spindle assembly in Eg5 inhibitor-treated cells with knocked down or exogenous HSP70 expression. HSP70-depleted or FLAG-tagged HSP70-overexpressing cells were treated for 1 h with TriC, which causes cells to exhibit a monopolar spindle^15,21^, or BRD, which enhances Eg5-dependent MT bundling and leads to abnormal bipolar spindles^21^. In the control cells, TriC treatment induced a monopolar spindle in 30% of mitotic cells; meanwhile, BRD treatment induced a specific type of abnormal bipolar spindle, manifested by the formation of numerous MT bundles, in ~45% of mitotic cells (Fig. 1d, and e, pLKO.1)^21^. However, in the HSP70-depleted cells, the proportion of mitotic cells with TriC-induced monopolar spindles was increased to 45%, and the proportion with BRD-induced abnormal bipolar spindles was increased to 65% (Fig. 1d and f, shHSP70), suggesting that loss of HSP70 enhanced the induction of spindle abnormalities by Eg5 inhibitors.

We then examined the effects of Eg5 inhibitors on spindles in FLAG-tagged HSP70-overexpressing cells. The TriC-induced monopolar spindles and BRD-induced abnormal bipolar spindles were both present in about 30% of mitotic cells harboring pFB-Neo, and the frequency of each abnormality was decreased to roughly 20% in FLAG-HSP70-WT-expressing mitotic cells (Fig. 1d, g and h), suggesting that overexpression of HSP70 ameliorates Eg5 inhibitor-induced spindle abnormalities. To test whether HSP70 chaperone activity regulates the mitotic functions of Eg5, we examined cells that overexpressed HSP70 with mutations in ATPase or substrate-binding domains. Cells were made to overexpress FLAG-HSP70-K71E, an ATPase-dead mutant that retains the passive substrate-binding activity, FLAG-HSP70-E175S, a mutant that is locked in an ADP-bound conformation and therefore lacking both ATPase and substrate-releasing abilities, or FLAG-HSP70-GG, a mutant with A406G + V438G double mutations in the substrate-binding domain^11,53–56^. These HSP70 mutant-overexpressing cells were treated with Eg5 inhibitors and spindles were examined. We found that spindle defects were attenuated in cells expressing FLAG-HSP70-K71E, as the proportions of cells with TriC-induced monopolar spindles and BRD-induced abnormal bipolar spindles were below 20% and 25%, respectively (Fig. 1i). However, in FLAG-HSP70-E175S and -GG-expressing cells, the percentages of cells with TriC-induced monopolar spindles and BRD-induced abnormal bipolar spindles were not significantly different from those in pFB-Neo-harboring cells (Fig. 1j and k). Thus, we can conclude that HSP70 relies on ATPase-independent substrate-binding activity to relieve Eg5 inhibitor-induced spindle defects.

### HSP70 interacts with Eg5 and is required for proper Eg5 mitotic localization and function within the spindle

**Fig. 2:**
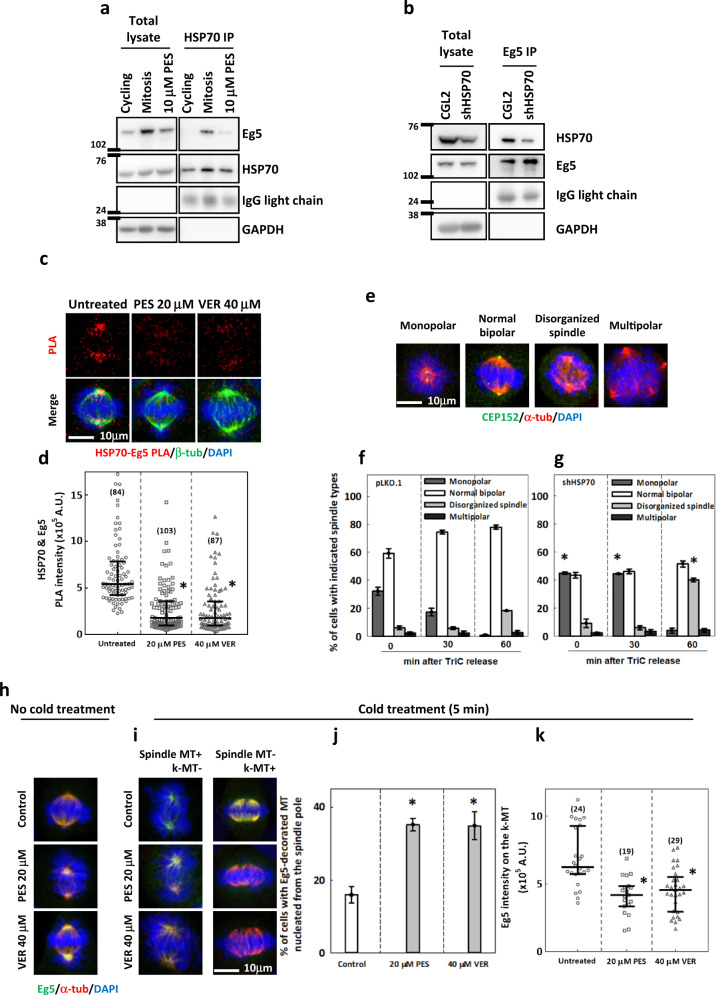
HSP70 interacts with Eg5 within the spindle and is required for proper Eg5 mitotic function and localization. **a** Eg5 was co-immunoprecipitated with HSP70 in the mitotic cells. The immunoprecipitation using HSP70 antibody were performed for extracts of CGL2 cells that were cycling, synchronized at mitosis or arrested at mitosis by 16 h PES treatment as described and Eg5 in the immunoprecipitates were detected by Western blotting. **b** Immunoprecipitation by Eg5 antibody was also performed using synchronized mitotic control or HSP70-depleted CGL2 cell extracts and HSP70 in the immunoprecipitates were detected by Western blotting. **c** A PLA was performed, and representative images are shown for HSP70-Eg5 PLA signal (red) with counterstaining of β-tubulin (β-tub, green) and DAPI (blue) in untreated and PES- or VER-treated CGL2 cells. **d** Relative intensity of HSP70-Eg5 PLA signal in the whole cell was measured as described. The scatter plot shows the interquartile distribution of HSP70-Eg5 PLA intensity, and the numbers in parentheses indicate the number of cells measured. *p < 0.05 compared to untreated by Mann–Whitney Rank Sum test. **e** Representative images of mitotic cells with the indicated types of spindle are shown after staining with CEP152 (green), α-tubulin (α-tub, red) and DAPI (blue). **f**–**g** Percentages of control (**f**, pLKO.1) and HSP70-depleted (**g**, shHSP70) mitotic cells with the indicated types of spindle. At least 600 mitotic cells were counted for each experiment. The mean ± SD from three independent experiments is shown. *p < 0.05 comparing shHSP70 to pLKO.1 by Student’s *t* test. **h** Representative images are shown for control and PES- or VER-treated mitotic cells fixed without cold treatment. **i** Representative images of the spindles in control and PES- or VER-treated cells that were fixed after 5 min cold treatment. Left, cells contained cold-resistant spindle MT with Eg5 colocalization (Spindle MT+; k-MT−). Right, cells with spindle MTs disassembled and k-MTs revealed (Spindle MT−; k-MT+). **j** The bar chart shows the percentages of mitotic cells with Eg5-decorated MTs nucleating from the spindle pole. At least 300 mitotic cells were counted for each experiment. The mean ± SD from three independent experiments is shown. *p < 0.05 compared to untreated by Student’s *t* test. **k** The scatter plot shows the interquartile distribution of relative Eg5 intensities on the k-MT, measured as described. The numbers in parentheses indicate the number of the cells measured. *p < 0.05 compared to untreated by Mann–Whitney Rank Sum test.

The effects of HSP70 on Eg5 inhibitor-induced spindle defects and cell death led us to suspect that HSP70 may interact with Eg5 during mitosis. By HSP70 antibody-mediated pull-down assay, we found that Eg5 is present in the immunoprecipitates of the synchronized mitotic but not cycling CGL2 cells (Fig. 2a). Importantly, Eg5 level was strongly reduced in the immunoprecipitates from the mitotic cells arrested by HSP70 inhibitor PES (Fig. 2a). Consistently, HSP70 was also present in the Eg5 immunoprecipitates from control mitotic cells but was present at a strongly reduced level from HSP70-depleted mitotic cells (Fig. 2b). These data suggested that HSP70 may associate with Eg5 during mitosis. We next examined the localization of HSP70-Eg5 complexes in the mitotic cells using a proximity ligation assay (PLA). The PLA signal for HSP70 and Eg5 was observed within the spindle, and the signal could be dramatically reduced by treating cells with PES or VER (Fig. 2c and d). Based on this localization, we tested whether HSP70 might be involved in antiparallel MT sliding, which is the main function of Eg5 that produces outward force required for separation of the spindle poles at mitosis onset and for maintaining the bipolarity of an already-formed spindle^4,25^. Since monopolar spindles were rarely observed in cells lacking HSP70 activity^10,11^, we reasoned that HSP70 might not be essential for spindle pole separation at mitosis onset and asked whether HSP70 is specifically involved in Eg5-dependent maintenance of spindle bipolarity. To answer this question, control and HSP70-depleted mitotic cells were treated with TriC for 1 h, followed by washout of the drug to allow the bipolar spindle reformation. In the control mitotic cells, TriC treatment induced monopolar spindles in 30% of the mitotic cells, while 60% of mitotic cells exhibited normal bipolar spindles. After TriC release, the percentage of cells with monopolar spindle was decreased to <15% at 30 min and close to 0% at 60 min, while those with normal bipolar spindles accounted for 75% at 30 min and 80% at 60 min (Fig. 2e and f, pLKO.1). This recovery suggested that reassembly of the bipolar spindle readily occurs after TriC washout. However, in the HSP70-depleted mitotic cells, TriC treatment induced monopolar spindles in 40% of the cells, while another 40% exhibited normal bipolar spindles; both percentages remained similar 30 min after TriC release, suggesting a delayed spindle reassembly process in the absence of HSP70 (Fig. 2e and g, shHSP70). At 60 min after TriC release, the proportion of cells with monopolar spindles was similar to that in control cells, but nearly 40% of the mitotic cells exhibited an abnormal bipolar spindle with disorganized MT networks (Fig. 2e ‘disorganized spindle′ and g), suggesting that HSP70-depleted cells have inaccurate bipolar spindle reassembly. These data indicated that HSP70 might not be essential for Eg5-mediated initiation of spindle pole separation during mitosis onset, but it is required for Eg5-dependent maintenance of bipolarity once the spindle has been established.

In yeast and mammals, the localization of Eg5 homologues to the spindle pole promotes MT crosslinking and capturing, while Eg5 binding to k-MT controls k-MT stability to facilitate chromosome congression^4,37–39,47,57^. To understand whether HSP70 regulates these Eg5 functions, we subjected control or HSP70 inhibitor-treated cells to cold treatment, a procedure that disassembles the spindle MT and reveals the k-MT, then examined Eg5 localization in different MT pools. In control metaphase cells without cold treatment, Eg5 was almost completely distributed to the spindle but became strongly accumulated at the spindle pole in the PES- and VER-treated cells (Fig. 2h). Five min cold treatment disassembled the spindle MT in the majority of the control cells while leaving ~16% of those with spindle MT retained (on which Eg5 was localized); however, the percentages of the PES- and VER-treated cells with Eg5-decorated spindle MT after 5 min cold treatment were increased to 40% (Fig. 2i, left column and j). These observations suggested that HSP70 inhibition led to spindle MT stabilization and retention of Eg5 at the stabilized spindle MT. Importantly, in the cold-treated cells in which the spindle MT was disassembled and the k-MT was revealed (Fig. 2i, right column), Eg5 intensity on the k-MT was significantly reduced in PES- and VER-treated cells compared to control cells (Fig. 2k), suggesting that HSP70 is required for k-MT localization of Eg5. Together, these data suggest that the HSP70 interaction with Eg5 is required for proper Eg5 localization and function in bipolar spindle assembly.

### HSP70 inactivation enhances accumulation of Eg5 at the spindle pole and prevents its inward movement along the spindle

**Fig. 3:**
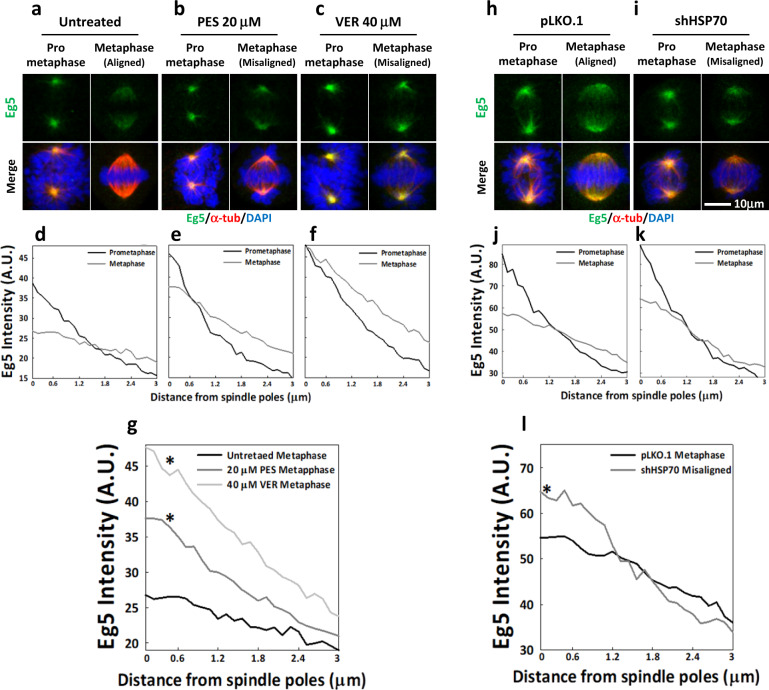
HSP70 inactivation enhances pole accumulation of Eg5 and prevents its inward movement. Representative images show the Eg5 (green) distribution within the spindle of untreated (**a**), PES-treated (**b**) and VER-treated (**c**) prometaphase and metaphase cells counterstained with α-tubulin (red) and DAPI (blue). Line-profile analysis of Eg5 intensity was performed as described. Eg5 intensity distributions were measured from at least 50 MT fibers in either prometaphase or metaphase cells. Data from cells imaged in two independent experiments were averaged to obtain the mean distribution curve and the 3 μm distance starting from spindle poles was plotted in (**d**) for untreated cells and in (**e**) and (**f**) for PES- and VER-treated cells, respectively. **g** Mean Eg5 distribution curves in untreated, PES- and VER-treated metaphase cells were plotted. Pairwise comparisons of the distribution curves were performed as described. *p < 0.05 compared to untreated. **h**–**k** Representative images show the Eg5 distribution within the spindle of the control (**h**. pLKO.1) and HSP70-depleted (**i**, shHSP70) prometaphase and metaphase cells. Eg5 intensity distributions were measured from at least 50 MT fibers in either prometaphase or metaphase cells. Data from cells imaged in two independent experiments were averaged to obtain the mean distribution curve and the 3 μm distance starting from spindle poles was plotted in (**j**) for control cells and in (**k**) for HSP70-depleted cells. **l** Mean Eg5 distribution curves in pLKO.1 control and HSP70-depleted metaphase cells were plotted. *p < 0.05 compared to pLKO.1.

After finding that HSP70 affects the localization of Eg5, we performed a detailed characterization of Eg5 localization dynamics in the presence or absence of HSP70 activity during mitosis progression. In untreated mitotic cells, Eg5 readily accumulated at the spindle pole during prometaphase and the signal spread inward, distributing uniformly along the spindle when the chromosomes were properly aligned during metaphase (Fig. 3a). However, in mitotic cells treated with HSP70 inhibitors, PES and VER, the chromosomes do not properly align at the metaphase plate^10,12^. In PES- and VER-treated cells, Eg5 accumulated at the spindle pole in prometaphase cells, and this accumulation persisted in metaphase cells with misaligned chromosomes (Fig. 3b, c). Line profiling analysis of Eg5 signal intensities in the untreated cells revealed that the mean Eg5 distribution exhibited a sharp descent along the spindle MT in prometaphase cells, with the signal almost completely absent 3 μm from the spindle pole; however, the descent in metaphase cells was much more moderate (Fig. 3d), suggesting that Eg5 accumulated at the poles during prometaphase but moved inward and toward the metaphase plate during metaphase. On the other hand, the mean Eg5 distribution curve in the PES- and VER-treated mitotic cells exhibited a sharp descent in both prometaphase cells and metaphase cells (Fig. 3e and f), suggesting that Eg5 accumulation at the poles persisted from prometaphase to metaphase. We further found that the mean Eg5 distribution curve exhibited a significantly sharper descent in PES- and VER-treated metaphase cells than in untreated metaphase cells (Fig. 3g). These data suggest that HSP70 inhibition enhanced Eg5 pole accumulation and disrupted its inward spread during metaphase. We also compared Eg5 distribution in the control and HSP70-depleted cells. As expected, Eg5 accumulated at the spindle poles during prometaphase and spread inward during metaphase in the control cells. In the HSP70-depleted cells, Eg5 exhibited significant pole accumulation in prometaphase and in metaphase cells with misaligned chromosomes, although the metaphase accumulation at the pole was not as apparent as in the inhibitor-treated cells (Fig. 3h–k). Comparisons between control and HSP70-depleted metaphase cells revealed a significantly sharper descent in Eg5 distribution curve in the depleted cells (Fig. 3l), supporting the notion that loss of HSP70 activity dramatically disrupts Eg5 inward spread during metaphase. Together, these data indicate a requirement for HSP70 to allow Eg5 inward spread along the spindle and chromosome congression during metaphase.

### HSP70 may modulate Eg5 oligomeric assembly to control Eg5 localization in the spindle

**Fig. 4:**
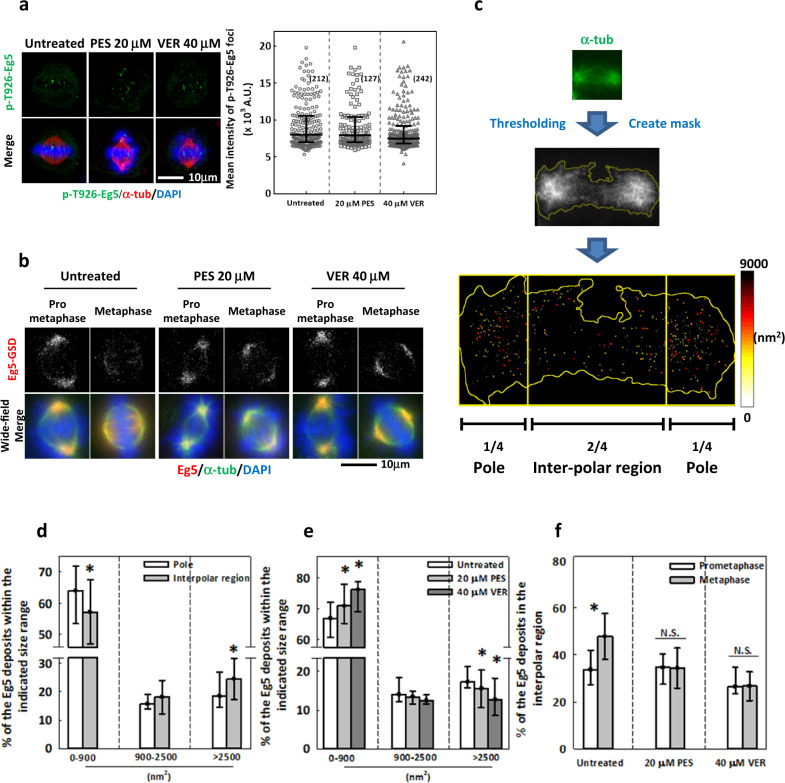
HSP70 may modulate Eg5 oligomeric assembly to control its localization in the spindle. **a** (left) Representative images show p-T926-Eg5 immunostaining (green) in untreated and PES- or VER-treated metaphase cells counterstained with α-tubulin (red) and DAPI (blue). (Right) Interquartile distribution of relative intensity of p-T926-Eg5 in the chromosome region was measured. Numbers in parentheses indicate the number of cells measured. **b** Representative GSD images show Eg5 immunostaining with corresponding wide-field images showing α-tubulin (green) and DAPI (blue) in untreated and PES- or VER-treated prometaphase and metaphase cells. **c** (Upper) The schematic illustration depicts the creation of a spindle mask by thresholding as described. (Lower) The Eg5 clusters were color-coded according to their sizes to reveal their distributions within spindle regions. **d** The percentages indicate Eg5 clusters within each indicated size range localizing to pole or inter-polar regions. The bar chart shows median ± 25th percentiles from at least 30 cells in four independent experiments. *p < 0.05 comparing inter-polar region to pole region by Student’s *t* test. **e** The percentages indicate Eg5 clusters within each indicated size range in the whole spindle of the untreated and PES- or VER-treated cells. The bar chart shows median ± 25th percentiles from at least 60 cells in four independent experiments. *p < 0.05 compared to untreated by Mann–Whitney Rank Sum test. **f** The percentages indicate the Eg5 clusters in the inter-polar regions of untreated and PES- or VER-treated prometaphase or metaphase cells. The bar chart shows median ± 25th percentiles from at least 20 cells in three independent experiments. *p < 0.05 comparing metaphase to prometaphase by Mann–Whitney Rank Sum test; N.S. no significance.

In order to infer the mechanism underlying HSP70 regulation of the timely inward spread of Eg5, we probed Thr-926-phosphorylated Eg5 (p-T926-Eg5); this posttranslational modification locates Eg5 to the kinetochore and regulates Eg5 association with spindle and kinetochore MTs^38,41,58^. We found that the mean intensity of p-T926-Eg5 on the kinetochores was not significantly changed by HSP70 inhibition (Fig. 4a), suggesting that HSP70 might not regulate Eg5 localization via its phosphorylation states. Single molecule assays performed in yeast revealed that Eg5 displays bi-directionality on MTs according to its oligomeric assembly; as such, a single Eg5 tetramer shows minus end-directed motility, while its coupling into tetramer teams at the minus ends triggers a switch to plus end-directed motility^45,47^. To see if HSP70 might regulate Eg5 oligomerization, we examined cells with immunostained Eg5 using GSDIM. This technique is a type of single molecule-based superresolution microscopy that allows us to measure the size and distribution of Eg5 molecules as a means to infer whether it occurs in oligomeric ensembles. From the GSD images of the untreated cells, we saw that Eg5 appeared as numerous clusters concentrated toward the spindle poles during prometaphase and spread inward during metaphase (Fig. 4b, Untreated). In the GSD images of the PES- or VER-treated cells, Eg5 clusters concentrated at the spindle pole in both prometaphase and metaphase cells (Fig. 4b, PES and VER). These subcellular patterns were entirely consistent with the results from confocal images. For further analysis, we divided each mitotic spindle into the pole region and the inter-polar region based on the α-tubulin immunostaining and then measured the sizes and distribution of Eg5 clusters within these regions (Fig. 4c). We classified Eg5 clusters into three size ranges, including 0–900 nm^2^, 900–2500 nm^2^ and >2500 nm^2^. Interestingly, the percentage of Eg5 clusters in the 0–900 nm^2^ size range was significantly lower in the inter-polar region than in the pole region, while the percentage of clusters above 2500 nm^2^ was significantly higher in the inter-polar region than in the pole region (Fig. 4d). These results suggested that the inter-polar region contains a higher proportion of larger Eg5 oligomeric ensembles than in the pole regions, implying that Eg5 proteins in large ensembles tend to localize to inter-polar regions. We then examined Eg5 clusters in the whole spindle and found that the percentage of Eg5 clusters with the size range of 0–900 nm^2^ was significantly increased in PES- and VER-treated mitotic cells, while the percentage above 2500 nm^2^ was significantly reduced. These results suggest that HSP70 inhibition may prevent the formation of large Eg5 ensembles (Fig. 4e). Together with the Eg5 distribution revealed by confocal imaging, the GSDIM findings suggested that HSP70 inhibition may decrease the propensity of Eg5 to form larger ensembles, thus disrupting its inward spread and reducing the Eg5 content at inter-polar regions of the spindle during metaphase. In support of this idea, we also found that the mean percentage of Eg5 clusters in the inter-polar region during metaphase was significantly higher than during prometaphase (48.7% in metaphase and 33.9% in prometaphase). Moreover, this increase was abolished in the PES- and VER-treated cells (Fig. 4f). Thus, HSP70 appears to be required for proper oligomeric assembly of Eg5, which likely ensures its inward spread at metaphase.

### HSP70 regulates Eg5 interactions with TPX2, acetylated tubulin and α-tubulin

**Fig. 5:**
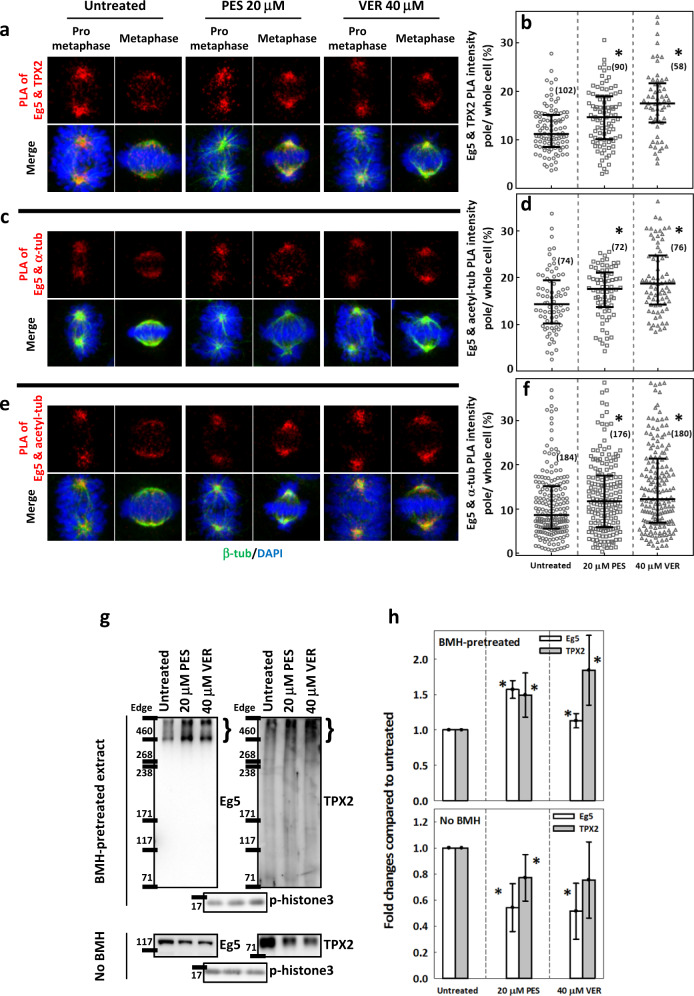
HSP70 is required for proper Eg5 interactions with TPX2, acetylated tubulin and α-tubulin. Representative images of Eg5-TPX2 PLA (**a**), Eg5-α-tubulin PLA (**c**) and Eg5-acetyl-tubulin PLA (**e**) signals are shown in red in untreated and PES- or VER-treated prometaphase and metaphase cells counterstained with β-tubulin (green) and DAPI (blue). The ratio of the PLA signal at the poles to the whole-cell signal was measured as described, and the interquartile distributions from two independent experiments are shown for (**b**) Eg5-TPX2, (**d**) Eg5-α-tubulin, and (**f**) Eg5-acetyl-tubulin. The numbers in parentheses indicate the number of the cells measured. *p < 0.05 compared to untreated by Mann–Whitney Rank Sum test. **g** (upper) BMH-mediated crosslinking was performed as described, revealing a protein complex >460 kD containing Eg5 and TPX2 in synchronized mitotic cells that were untreated or treated with PES or VER. (Lower) Total Eg5 and TPX2 proteins in the denatured lysate. **h** The bar charts show the levels of Eg5 and TPX2 compared to the untreated cells, as normalized by the mitosis marker p-histone3 for BMH-crosslinked samples (upper) and no BMH-treated samples (lower). Mean ± SD of six replicates from three independent experiments is shown. *p < 0.05 compared to untreated by Student’s *t* test.

The finding that HSP70 modulates Eg5 oligomeric assembly implies that HSP70 may control the assembly/disassembly of Eg5-containing protein complexes, which is consistent with reported chaperone functions of HSP70^59–61^. In addition to the intermolecular coupling of multiple Eg5 tetramers, it has also been proposed invertebrate and mammalian cells that Eg5 motility can be controlled by its interaction with TPX2. TPX2 interacts with both Eg5 and MT and thus targets Eg5 to the spindle, restricts Eg5 motility on MT and stabilizes spindle and kinetochore MTs together with Eg5^37,49–51^. Since HSP70 inactivation simultaneously disrupted Eg5 localization and MT dynamics (Fig. 2g–i), we further probed the Eg5-TPX2 interaction in cells with or without HSP70 activities by PLA. Strikingly, the PLA signal appeared at the spindle pole during prometaphase and became evenly distributed along the spindle during metaphase. In contrast, PLA signal in the PES- and VER-treated mitotic cells persistently appeared at the spindle pole in both prometaphase cells and metaphase cells (Fig. 5a), nearly identical to Eg5 immunostaining patterns (Fig. 3a–c). The fraction of PLA signal at the pole (normalized to the signal in the whole cell) was significantly increased in the PES- and VER-treated cells (Fig. 5b). Based on previous reports that TPX2 restricts Eg5 plus end-directed motility in vitro^50^, this finding suggested that HSP70 inactivation may enhance the Eg5-TPX2 interaction at the spindle pole, preventing Eg5 from moving inward. Since the Eg5-TPX2 interaction contributes to MT stabilization^50^ and HSP70 inhibition restrains Eg5 to the cold-resistant spindle pole MT in cold-treated cells (Fig. 2i), we then performed a PLA for Eg5 and acetylated tubulin, a marker of the stabilized MT^62^. A PLA for Eg5 and α-tubulin was also conducted as a control experiment. Both Eg5-acetylated tubulin and Eg5-α-tubulin PLA signals in untreated cells exhibited pole accumulation in prometaphase and inward spread in metaphase, while PES- and VER-treated cells exhibited persistent pole accumulation in prometaphase and metaphase (Fig. 5c and e). For both Eg5-acetylated tubulin and Eg5-α-tubulin PLA, the fractions of signal at the pole were significantly increased by PES and VER treatments (Fig. 5d and f), further supporting the idea that HSP70 inactivation restricts Eg5 to the pole, thereby inducing Eg5-dependent MT stabilization at the spindle pole.

To perform another measure of Eg5-TPX2 complex formation, we harvested synchronized mitotic cells and treated the cell homogenates with BMH, a homobifunctional maleimide crosslinker for sulfhydryl groups, to stabilize the Eg5-containing protein complexes followed by western blotting analysis. We found that nearly all Eg5 proteins were incorporated into large complexes of more than 460 kD, which approximately corresponds to the size of an Eg5 homotetramer (Fig. 5g, upper left). Therefore, this result is consistent with a scenario in which Eg5 tetramers assemble into a larger protein complex. Importantly, TPX2 also appeared at the same molecular weight range in BMH-crosslinked extracts (Fig. 5g, upper right). In addition, the levels of both Eg5 and TPX2 in the protein complexes were significantly higher in the PES- or VER-treated mitotic cell extracts than untreated (Fig. 5h), lending further evidence to the idea that HSP70 inactivation may enhance Eg5-TPX2 interaction to restrict Eg5 at the spindle poles and reduce its inward movement. These data and the GSD images collectively indicate that HSP70 is required for proper protein complex formation and the interaction between Eg5 tetramers or Eg5 and TPX2 to control inward spread of Eg5 during metaphase.

### The ATP-independent substrate-binding activity of HSP70 is required for Eg5 inward spread and chromosome congression

**Fig. 6:**
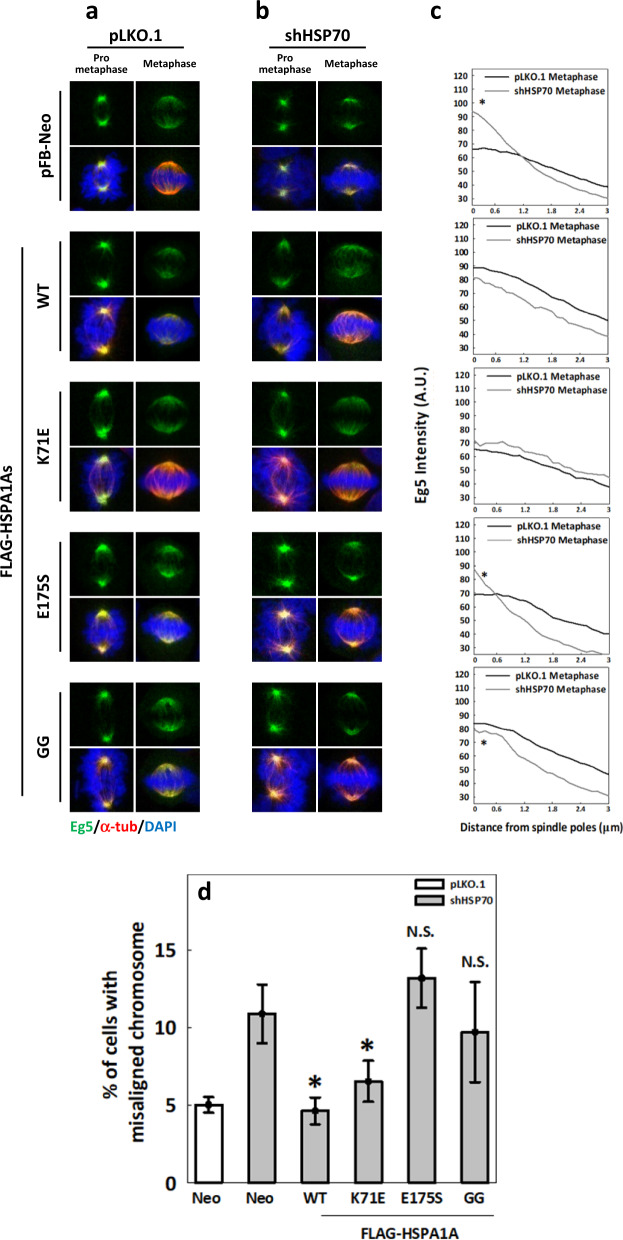
The ATP-independent substrate-binding activity of HSP70 is required for Eg5 inward movement and chromosome congression. Cells harboring pFB-Neo empty vector or expressing WT or mutant FLAG-HSPA1As were transiently depleted of endogenous HSP70. Representative images show Eg5 (green) distribution at prometaphase and metaphase in these cells for (**a**) knock-down control pLKO.1 and (**b**) HSP70 depletion. The mean Eg5 distribution curves at metaphase were obtained from at least 30 MT fibers and are shown for (**c**) pLKO.1 and shHPS70. *p < 0.05 comparing shHSP70 to pLKO.1. **d** Percentages of cells with the indicted expression cassettes with misaligned chromosome during metaphase are shown. At least 600 mitotic cells were counted for each experiment. The mean ± SD from three independent experiments is shown. *p < 0.05 compared to Neo-shHSP70; N.S. no significance.

The finding that HSP70 modulates complex formation between Eg5 tetramers and TPX2 implies an involvement of HSP70 chaperone activity in the regulation of Eg5. To determine whether HSP70 substrate binding regulates Eg5 function, cells stably expressing FLAG-HSP70-K71E, FLAG-HSP70-E175S, and FLAG-HSP70-GG were depleted of endogenous HSP70^11^, and Eg5 distribution within the spindle was examined. In cells harboring pFB-Neo empty vector or stably expressing each HSP70 construct, Eg5 was distributed in an expected manner, with pole accumulation at prometaphase and inward spread at metaphase (Fig. 6a). After depletion of endogenous HSP70 in these cells (Fig. 6b), persistent pole accumulation of Eg5 at both prometaphase and metaphase was observed in cells harboring pFB-Neo, FLAG-HSP70-E175S, and FLAG-HSP70-GG constructs but not in those harboring FLAG-HSP70-WT and -K71E constructs. Comparing the mean Eg5 distribution curve at metaphase in endogenous HSP70-depleted cells to that in the control cells revealed a sharper descent in occupancy moving away from the poles in cells harboring pFB-Neo or FLAG-HSP70-E175S and -GG, but not in -WT and -K71E (Fig. 6c). Thus, HSP70 depletion-induced defects in Eg5 distribution are rescued by the expression of FLAG-HSP70-WT and -K71E but not -E175S and -GG. These distribution rescue effects mirror those seen for Eg5 inhibitor-induced spindle defects (Fig. 1g–k). Since we previously reported HSP70 depletion or inhibition causes chromosome misalignment^10^ and the disruption of Eg5 inward spread was found to largely coincide with chromosome misalignment in HSP70-deficient cells (Fig. 3), we further examined the effects of FLAG-HSP70-K71E, -E175S and -GG on HSP70 depletion-induced chromosome misalignment. Accordingly, expression of FLAG-HSP70-WT and -K71E but not -E175S and -GG rescued HSP70 depletion-induced chromosome misalignment (Fig. 6d). Collectively, these data indicate that ATP-independent substrate-binding activity of HSP70 is required for Eg5 inward spread and chromosome congression during metaphase.

## Discussion

HSP70 is a highly conserved inducible molecular chaperone essential for cellular proteostasis; it has been shown to be targeted to the mitotic centrosome by multiple mitotic kinases^11,12,63–65^, and its pharmacological inhibition disrupts mitotic spindle assembly to induce mitotic arrest and mitotic cell death^10,11^. Eg5 is a kinesin MT motor that plays an essential role in spindle assembly, making it a promising target for antimitotic cancer therapeutics^4,14,25^. However, the clinical application of Eg5 inhibitors has been impeded by development of drug resistance in cancer cells and suboptimal results in clinical trials^14,23,24^. In this study, we found that HSP70 expression and activity oppose cellular chemosensitivity to Eg5 inhibitors, suggesting a possible role of HSP70 in resistance to the Eg5 inhibitors.

In our results, Eg5 and HSP70 were co-immunoprecipitated by each other in mitotic cells and colocalization of Eg5 and HSP70 was observed within the mitotic spindle, indicating a direct interaction between HSP70 and Eg5 during mitosis. This interaction was expected based on the results of a systematic pull-down assay in yeast that showed the interaction network for HSP70 includes Eg5 homologous proteins^52^. In addition, HSP70 inactivation did not induce the formation of monopolar spindles, as was observed after Eg5 inhibition by TriC^15,21^. Instead, our experiments examining bipolar spindle reassembly after TriC washout revealed that HSP70 is required for Eg5-dependent maintenance of spindle integrity. Interestingly, we also found that HSP70 ameliorated the respective defects induced by TriC and BRD, two Eg5 inhibitors with distinct modes of action^20,21^, suggesting that HSP70 may modulate multiple functions of Eg5 during mitosis rather than only one. Collectively, these data confirmed that the HSP70 interaction with Eg5 is required for the mitotic functions of Eg5, thus explaining the enhanced cytotoxic effects of combined inhibition of HSP70 and Eg5.

A bipolar homotetrameric conformation of Eg5 was revealed by cryogenic electron microscopy, and loss of Eg5 function leads to formation of a monopolar spindle with unseparated spindle poles; this pair of observations led researchers to conclude that Eg5 acts on antiparallel MTs at the spindle midzone^15,27,29^. Intriguingly, the physiological localization of Eg5 is enriched near the spindle pole, where the spindle is mainly comprised of parallel MTs^4,25,26^. Studies on invertebrate, vertebrate, and mammalian cells as well as in vitro systems demonstrated that different pools of Eg5 may exist within the spindle, with each pool playing different roles during spindle assembly. Beyond its function in sliding apart antiparallel MTs, Eg5 appearing near the spindle pole region may facilitate crosslinking to stabilize parallel MTs during mitosis^21,35,36^; furthermore, Eg5 near the kinetochore controls k-MT growth rate and stability, an action that is required for accurate k-MT attachment and chromosome congression^37–39^. Thus, a precise distribution of Eg5 pools within the spindle is critical for the spatial control of MT dynamics during spindle assembly. We observed that Eg5 accumulates at the spindle pole during prometaphase and inwardly spreads within the spindle at metaphase, a phenomenon also observed in other studies^51,66,67^, which indicates the existence of a cell-intrinsic spatiotemporal control system for Eg5 distribution. Importantly, our results showed that HSP70 inhibition or depletion disrupted the inward spread of Eg5 during metaphase and enhanced its spindle pole accumulation. This disruption was accompanied by the occurrence of spindle defects and chromosome misalignment. Since we previously utilized cold treatment to demonstrate that HSP70-deficiency induces stabilization (and thus cold-resistance) of the spindle pole MT^10^, we used a similar approach in the current study to find that HSP70 inhibition in mitotic cells retained Eg5 on the cold-resistant spindle pole MTs and reduced Eg5 localization to k-MTs. These findings presumably explain the previous reports of spindle pole MT stabilization, k-MT destabilization, and misalignment of the chromosomes in cells lacking HSP70 function^10,12^. Based on our current findings and these earlier studies, we therefore conclude that HSP70 is required for Eg5 inward spread at metaphase to ensure the localization of Eg5 to k-MTs, and this function is important for spatiotemporal accuracy of MT dynamics and integrity of the spindle. Inaccurate distribution of Eg5 resulting from HSP70 deficiency may thus deregulate MT dynamics, leading to spindle defects and chromosome misalignment.

While the differential localization of Eg5 within the spindle at different mitotic stages implies an intrinsic control system for Eg5 distribution on MTs, the mechanism of this regulation in mammalian mitotic cells remains elusive and can only be conjectured based on studies in various model organisms and in vitro systems. CDK1-mediated phosphorylation of Eg5 at T926 and its equivalent sites in yeast, flies and xenopus was shown to modulate Eg5 association with MTs or MAPs and may thus control Eg5 localization to the spindle MTs or k-MTs^29,40,41,58,68^. However, HSP70 probably does not regulate Eg5 function by controlling the state of Eg5 CDK1-dependent phosphorylation, as evidenced by our observation that p-T926-Eg5 immunostaining did not changed after HSP70 inhibition. On the contrary, our data are in better agreement with another working model, wherein HSP70 may control Eg5 tetramer oligomerization or Eg5-TPX2 interactions to regulate Eg5 distribution. This model takes into consideration the previous findings in yeast, vertebrate, and mammalian cells that Eg5 motility or directionality can be controlled by its oligomerization or interactions with other regulatory proteins, such as TPX2 or dynein^37,44–51^. For example, an in vitro single molecule assay was used to demonstrate that Cin8, the Eg5 homologue in yeast, displays bi-directionality on MTs that is regulated by its oligomeric states; i.e., a single Cin8 tetramer exhibits minus end-directed motility and thus tends to accumulate at the minus end, whereas the intermolecular coupling between Cin8 tetramers into an ensemble induces a switch to plus end-directed motility^45,47^. Our study of BMH-mediated protein crosslinking showed that Eg5 in mitotic cells appeared in a molecular weight range of >460 kD, indicating that Eg5 may function as oligomerized tetramers and/or tetramers complexed with accessory proteins during mitosis. In addition, our GSD images showed that larger Eg5 ensembles tend to localize to the inter-polar region while smaller Eg5 ensembles tend to accumulate at the pole region, implying a similar regulation of Eg5 motility occurs in human cells as observed in yeast. Moreover, HSP70 inhibition significantly reduced the tendencies of Eg5 to form large ensembles, disrupted their localization to inter-polar region, and led to the elevated accumulation of Eg5 in the BMH-crosslinked high-molecular-weight complex (Figs. 4e, f, and 5g). Thus, these findings lead us to hypothesize that HSP70 may be required for proper oligomerization of Eg5 tetramers at the spindle pole, which would be expected to promote Eg5 plus end-directed motility on MT to ensure its inward spread at metaphase.

Studies in in vitro systems or invertebrate, mammalian and human cells demonstrated that TPX2, a RAN-regulated spindle assembly factor, targets Eg5 to the spindle pole, while stabilizing MTs with Eg5 and restricting plus end-directed motility of Eg5 on MTs^37,50^. Importantly, our PLA results showed that Eg5 and TPX2 colocalize at the spindle pole during prometaphase, and a previous study indicated that Eg5, but not TPX2, localizes to the spindle midzone during metaphase^66^. Together, these findings suggest that TPX2 may act as a brake for Eg5 motor activity, and Eg5 may disengage from TPX2 during metaphase in order to move inward. Our finding that HSP70 inactivation enhanced the Eg5-TPX2 interaction at the spindle pole and led to TPX2 accumulation in the BMH-crosslinked high-molecular-weight complex (Fig. 5a, b, g and h) allows us to further hypothesize that HSP70 may function to release Eg5 from TPX2, thus enabling Eg5 inward movement at metaphase. Interestingly, Eg5 ensembles were occasionally found to split apart and reassemble, and protein complexes containing Eg5 and TPX2 or other regulatory proteins were found to be dynamically reorganized^47,51^. Therefore, Eg5-containing proteins complex may undergo continuous assembly/disassembly as a result of intricate proteostasis regulation. Since HSP70, Eg5 and TPX2 all localized to the spindle pole during prometaphase, our data suggest that HSP70, a known master regulator of proteostasis, could coordinate Eg5-Eg5 or Eg5-TPX2 interactions at the spindle pole to regulate Eg5 motility. In future studies, an in vitro motor gliding assay would be helpful to test these hypotheses.

Regulation of the assembly/disassembly of protein complexes is a major HSP70 chaperone activity^61^. In *C. elegans* and mammalian cells, HSP70 in the ER has been reported to stabilize nascent ERG peptides, maintain their proper folding and prevent them from degradation before their assembly into a functional tetrameric ERG-type K^+^ channels^59^. In addition, ribosome-associated HSP70 in yeast engages the newly synthesized interacting domain of hetero-oligomeric client proteins to prevent premature inter-domain association, and it dissociates with clients before the onset of accurate subunit association^69^. Thus, it was proposed that HSP70 may facilitate protein complex assembly by transiently binding to client proteins at their aggregation-prone interacting subunits; this action stabilizes the client proteins and prevents inaccurate subunit association, misfolding or degradation before their correct assembly into a functional complex^61^. Our HSP70-Eg5 PLA results showed that HSP70 inhibition in mitotic cells disrupted the binding between HSP70 and Eg5 (Fig. 2b), which may prevent the oligomerization of Eg5 tetramers. Accordingly, the results of our BMH-mediated crosslinking experiments showed that Eg5 tetramer complexes were significantly increased in the extracts of PES- or VER-treated mitotic cells, whereas the total level of Eg5 protein slightly decreased compared to untreated cell extracts. Considering the previous report that Eg5 level is controlled by proteasome-mediated degradation^70^, our data thus imply that compromised HSP70 activity may deregulate the assembly of Eg5-containing protein complexes, destabilize Eg5 proteins and result in Eg5 degradation. Surprisingly, FLAG-HSP70-K71E was found to rescue Eg5 functions in HSP70-depleted or Eg5 inhibitor-treated cells to levels comparable with FLAG-HSP70-WT, suggesting that the chaperone activities retained in this mutant are sufficient to maintain Eg5 distribution within the spindles. Since the K71E mutation significantly disrupts ATP binding and hydrolysis but behaves like WT-HSP70 in terms of its association/dissociation with client proteins^53,54^, this mutant may passively prevent client proteins from misfolding or aggregation, a mode of action that has been proposed based on an in vitro study of nucleotide-free HSP70^5^. Together, our findings are consistent with a scenario in which HSP70 transiently associates with Eg5 during prometaphase to passively prevent misfolding or aggregation, thus stabilizing Eg5 protein and allowing appropriate homo- or hetero-oligomerization and motility on MTs.

Notably, the functions of Eg5 may also require other aspects of regulations. For example, Eg5 needs to be accurately recruited to the spindle pole at the mitosis onset^38,49^, which would be speculated to depend on the properly functioning mitotic centrosome. In our previous report^11^, we have demonstrated that the K71E mutant partially rescued the defects of the mitotic centrosome in HSP70-depleted cells, implying that the passive-binding activity may support but not suffice for accurate mitotic centrosome assembly. Since the E175S showed no rescue effects in HSP70-depleted cells, we speculated that the HSP70 ATPase cycle, which is facilitated by the co-chaperones such as HSP40 and NEFs, may be required for accurate assembly of a fully functional mitotic centrosome that is responsible for recruiting massive mitotic regulators, including Eg5. Therefore, co-chaperone-assisted HSP70 ATPase cycle may play roles in Eg5 recruitment to the centrosome or in other Eg5 functions.

In conclusion, we showed that the chaperone activity of HSP70 is required for the accurate spatiotemporal control of Eg5 localization and function during spindle assembly, and this regulation can account for the HSP70 inhibition-mediated enhancement of Eg5 inhibitor cytotoxicity in proliferating cells. Notably, our study focused on the cytoprotective functions of the intracellular HSP70, whereas the cytoprotective functions of the extracellular HSP70 have also been documented^71^. The potential roles of extracellular/circulating HSP70 in mitotic spindle assembly and mitosis progression warrant further investigation. Given the importance of both Eg5 and HSP70 in spindle assembly and that their overexpression has been observed in cancer cells, our results suggest that HSP70 inactivation may improve the therapeutic effects of current Eg5 inhibitors.
